# Dose Articulation in Preclinical and Clinical Stroke Recovery: Refining a Discovery Research Pipeline and Presenting a Scoping Review Protocol

**DOI:** 10.3389/fneur.2019.01148

**Published:** 2019-11-06

**Authors:** Emily Dalton, Leonid Churilov, Natasha A. Lannin, Dale Corbett, Kathryn S. Hayward

**Affiliations:** ^1^Department of Physiotherapy, Melbourne School of Health Sciences, University of Melbourne, Parkville, VIC, Australia; ^2^Melbourne Medical School, University of Melbourne, Parkville, VIC, Australia; ^3^NHMRC CRE in Stroke Rehabilitation and Brain Recovery, University of Melbourne, Heidelberg, VIC, Australia; ^4^Department of Neurosciences, Central Clinical School, Monash University, Melbourne, VIC, Australia; ^5^Department of Occupational Therapy, Alfred Health, Prahran, VIC, Australia; ^6^Cellular and Molecular Medicine and Canadian Partnership for Stroke Recovery, University of Ottawa, Ottawa, ON, Canada; ^7^AVERT Early Rehabilitation Research Group, Stroke Theme, Florey Institute of Neuroscience and Mental Health, University of Melbourne, Heidelberg, VIC, Australia

**Keywords:** stroke rehabilitation, treatment dose, clinical trial, translational medical research, animal model

## Abstract

**Introduction:** Despite an increase in the quality of clinical trials in stroke recovery, interventions have failed to markedly impact the trajectory of recovery after stroke. Failure may be due to the lack of consideration for the complexity of dose and its articulation within research trials. Prior to commencing the scoping review, we identified two research gaps to be addressed. Firstly, transparent application of a multidimensional definition of dose to clinical trial phases and secondly, the development of a quality tool to critique the articulation of dose across the pipeline. Building on this, we present the protocol for a scoping review that aims to synthesis what is known about dose articulation in stroke recovery in preclinical and clinical populations, and characterize research designs and statistical approaches used in dose articulation trials, and the associated advantages and disadvantages.

**Methods:** The scoping review will apply Arksey and O'Malley's methodological framework. Two systematic searches that target preclinical and clinical literature will be run in Medline and Embase, which will be complimented by consultation with field experts and hand searching of included trials and relevant reviews. Search results will be imported into Covidence for transparent management. One reviewer will screen all abstracts and titles. Two reviewers will screen full text and a third reviewer included to resolve discrepancies. A standardized data charting form will be used to extract information and appraise the intervention description, risk of bias, and quality of both preclinical and clinical studies. Results will be summarized in tabular and narrative format to inform the development of recommendations for future research. Ethics approval is not required as data used will be secondary and de-identified.

**Conclusion:** Development of a new quality tool to appraise the quality of both preclinical and clinical dose studies may serve to strengthen collaborative efforts between the fields. The findings from this review will advance the use of a discovery pipeline in stroke recovery research to ultimately inform clinical practice.

## Introduction

Delivery of the right dose of rehabilitation, at the right time, to the right person continues to challenge preclinical and clinical stroke recovery researchers, as well as clinicians (1). The increase in stroke recovery research across behavioral therapy domains has seen a noticeable shift toward Phase III clinical trials (2, 3). Appropriate application of this phase of clinical trial can elicit meaningful results through appropriately powered studies that are built upon iteratively modeled intervention development (4). Unfortunately, there has not been a concomitant improvement in the recovery trajectory for stroke survivors. The search for a game-changing stroke recovery intervention that sets the field on a new path, remains a goal for the research, and clinical community (1, 5).

The international Stroke Recovery and Rehabilitation Roundtable (SRRR) was recently convened to provide consensus in key areas including development, conduct, and reporting of stroke recovery research (6). The SRRR highlights the lack of consideration for the complexity of dose as a potential reason for neutral, Phase III trial results (5). Compared to pharmaceutical interventions, the construct of therapy dose in stroke recovery trials extends far beyond the notion of total amount of therapy. Therapy dose is multifaceted with a clear need for individual constructs of dose, including time, scheduling, and intensity, to be articulated and understood. As a way to address this challenge, SRRR provides a consensus recommendation to implement, a systematic discovery pipeline to translate preclinical experimental findings through to phased clinical trials where the results of prior studies directly influence the conduct of later studies (5, 7). A similar approach has been successfully implemented to support the advancement of acute stroke therapies (8–10). The complexity of rehabilitation interventions (and dose) needs to be considered to successfully adopt this approach in stroke recovery.

### Study Rationale and Hypothesis

In this paper, we provide a narrative overview of why the failure to consider therapy dose (11) and the poor implementation of a systematic discovery research pipeline could be preventing development of breakthrough interventions for stroke recovery (1, 6). This is followed by the protocol for our systematic scoping review. The review design was chosen to broadly map current research, identify research gaps, and highlight opportunities for future research. The specific question of the scoping review is: *What is known about dose articulation in stroke recovery in both preclinical and clinical populations?* The specific aims of the scoping review are to:

1. Synthesize the literature on dose articulation in preclinical stroke recovery research;
2. Synthesize the literature on dose articulation in clinical stroke recovery research; and
3. Characterize the literature on research designs and statistical approaches used in dose articulation trials, and the associated advantages and disadvantages.

**Figure 1:**
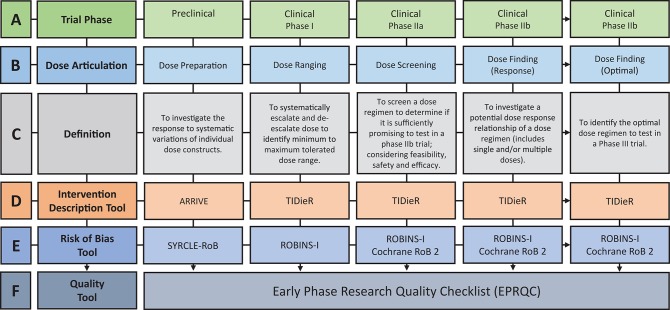
Overview of the discovery pipeline. **(A)** Trial Phase: *Preclinical*: Studies involving animal subjects. *Clinical*: Studies involving human participants (12). **(B)** Dose articulation: Type of dose articulation trial relevant to phase. Example studies for each phase: Preclinical—Dose Preparation (13, 14). Clinical—Dose Ranging (15, 16), Clinical—Dose Screening (17) Clinical—Dose Finding (Response) (18, 19), Clinical—Dose Finding (Optimal) (20). **(C)** Definition: Definition of how dose is articulated in each phase. **(D)** Intervention Description Tool: *ARRIVE*: Animal Research: Reporting of *in vivo* Experiments (21). *TIDieR*: Template for Intervention Description and Replication Checklist (22). **(E)** Risk of Bias Tool: *SYRCLE-RoB*: Systematic Review Center for Laboratory Animal Experimentation's Risk of Bias Tool (23). *Selection Bias*: Is the population representative of population being analyzed. *Cochrane RoB 2*: Cochrane Risk of Bias 2 (24). *ROBINS-I*: Risk of Bias in Non-Randomized Studies-of Interventions (25). **(F)** Quality Tool: *EPRQC*: Early Phase Research Quality Checklist adapted from quality assessment checklist for phase I cancer trials (26).

Within this review, dose articulation encompasses all aspects related to dose preparation, ranging, selection, or finding (see Figure 1); studies included in this review will address one or more of these aspects. We include interventions that target behavioral motor therapies in stroke recovery (e.g., upper limb, lower limb, and exercise rehabilitation) as this area has been studied more than any other across preclinical and clinical models of stroke (27, 28), and represents a well-established clinical need expressed by stroke survivors (29).

We hypothesize that preclinical and early phase, clinical trials in the area of dose articulation post stroke are rare. Studies will not have considered dose to be multidimensional, targeting time, scheduling, and intensity.

### Understanding the Complexity of Dose in Stroke Recovery Research

Dose is usually described in medical research as a unidimensional construct (e.g., quantifiable amount of an active ingredient known to influence a specific therapeutic target) that reflects the dose of a given drug that is effective, safe, and tolerable (30). In contrast, there is a breadth of definitions used to define dose in preclinical experiments and clinical trials in the field of stroke recovery, all of which highlight dose as an active, multidimensional ingredient (11, 31). Preclinical experiments have most commonly considered dose in terms of repetitions (e.g., number of reaches) and sessions per day. Clinical trials most often focus on time in therapy (minutes/hours), largely for pragmatic reasons despite not being an accurate measure of actual work performed in therapy (32). To effectively articulate dose we need to consider all constructs including *time* (in minutes or hours “on task”) (33), *repetitions* (of successful and unsuccessful movement e.g., wrist extension, or of function e.g., reaches of a cup) (4), *frequency* (number of sessions per day and per week e.g., 2 sessions per day, 5 days per week) (4, 33), *intensity* (e.g., rating of perceived exertion RPE, challenge point, and heart rate HR) (33), and *scheduling* (length of program e.g., 10 weeks) (4). The lack of a universal definition of dose that includes the above multidimensional constructs remains a barrier to moving research forward in this area. In this paper, dose is defined as having three main constructs which can be articulated or fixed within dose studies based on the research question asked. The three constructs are as follows:

- Time on task, in minutes or hours;
- Scheduling, number of sessions per day/per week and the length of the program; and
- Intensity, the level of difficulty assigned to a task e.g., repetitions per minute, rating of perceived exertion, or the set task challenge point.

The ambiguity of dose reporting in clinical trials is reflected across clinical practice guidelines globally (34–36). The 2017 Australian Stroke Foundation (35) guidelines are the most recently updated, and were chosen to demonstrate current dose reporting (37) however, guidelines published in 2016 in the United Kingdom (34) and 2017 in the United States (36) reflect a similar recommendation. Specifically, the Australian guidelines provide nine recommendations for behavioral motor interventions to address upper limb activity post stroke; only one has a strong recommendation (constraint induced movement therapy) to be offered to all eligible patients (35), and two provide information regarding the dose of therapy required. Interestingly, the strong recommendation includes a dose regimen of “a minimum of 2 h of active therapy per day for 2 weeks, plus restraint for a least 6 h a day” (35). The guidelines have a section, *amount of rehabilitation*, which provides a strong recommendation for rehabilitation to be “structured to provide as much scheduled therapy as possible” (35). This is supported by a weak recommendation for the dose to include “a minimum of 3 h a day of scheduled therapy” and “at least 2 h of active task practice” during that time (35). The ambiguity of dose reporting, challenges clinicians globally to operationalize dose recommendations into practice and supports the need for a discovery pipeline approach to systematic dose selection.

### A Discovery Pipeline to Guide Systematic Stroke Recovery Research

The implementation of a systematic pipeline is a well-recognized approach for the development and testing of new pharmaceuticals (12, 38), and is now a SRRR consensus recommendation to progress stroke recovery research forward (5, 7). The pipeline starts at the preclinical level with the focus to understand the biological mechanisms underlying recovery. Preclinical findings are then translated to early phase clinical trials to establish safety and dosage, prior to completing later phase clinical trials to determine efficacy and effectiveness. A published systematic review highlights that few stroke recovery, dose articulation studies currently implement a phased approach (11).

The translation of findings between preclinical and clinical research is a well-established challenge (7). Stroke Therapy Academic Industry Roundtable (STAIR) (8) was founded to address this challenge in the treatment of acute stroke (8). Since the initial publication in 1999, STAIR has supported the advancement of acute stroke science through the provision of systematic, consensus recommendations to advance the translation from preclinical to clinical research (9, 10). The international SRRR collaboration identified the translation of preclinical findings to clinical research as a priority area for stroke recovery research (6, 27). A resulting publication highlighted some of the current limitations of preclinical approaches, including the use of stroke models that do not capture the chronicity of impairment and heterogeneity of human stroke (7). Another important consideration is the use of preclinical findings to provide a biological rationale for a given intervention and chosen dose (11, 17). Currently, there is a lack of consistency within literature regarding translation of intervention and dose findings from preclinical to clinical stroke populations, as reflected in three recent, large randomized controlled trials (RCT) on motor rehabilitation after stroke (39–41). One trial broadly referenced preclinical and clinical research as a biological rationale for their intervention choice (41), while the other two trials referenced clinical research only (39, 40). What is missing from all these trials, however, is a clear rationale (i.e., preclinical and clinical dose articulation research) regarding the dose of the intervention to be tested. The shift to include preclinical literature in the rationale for clinical trial decision making is the first step in the discovery pipeline. The translation of dose articulation findings along the pipeline relies on preclinical experiments using suitable research designs, and clinical researchers systematically translating preclinical findings through the incremental steps of phased research. The steps of the discovery pipeline are visually represented in Figure 1A.

Preclinical experiments aim to articulate dose by investigating the response to systematic variations of individual dose constructs (Figures 1B,C). An example of the dose preparation process is the comparison of higher intensity training (2 × 15 min sessions) vs. lower intensity training (1 × 15 min session) vs. control (no training) on skilled motor performance (14). To translate these findings, early phase clinical trial designs should be implemented to test the dose range in humans. Early phase research includes Phase I and II trial designs which are most commonly used to test and develop new drugs (30). The Australian Government Therapeutic Goods Administration (TGA) (12) and the American Food and Drug Administration (FDA) (38) define the purpose of Phase I trials as “safety and dosage” and Phase II trials as “efficacy and safety.” Drawing on the purpose of these designs, dose articulation can be mapped to the incremental steps of early phase clinical research through the terminology of dose ranging, dose screening, and dose finding (Figures 1B,C). Dose ranging is considered a Phase I trial, which systematically escalates and de-escalates dose to indicate a minimum to maximum tolerated dose range (12). Published Phase I trials in stroke recovery have chosen one construct of dose (e.g., repetitions or time) to escalate and de-escalate (15, 16). As previously discussed, dose has multiple constructs and the articulation of only one of these constructs within early phase studies may impact the outcome of later stage intervention trials.

Phase IIa is defined as dose screening, aiming to determine if a dose regimen is sufficiently promising to test in a phase IIb trial which considers the feasibility, safety, and efficacy of a given dose (42). For example, a phase IIa trial would aim to determine the feasibility of completing 300 repetitions in a 1 h outpatient session with chronic stroke patients (17). If deemed feasible, the dose regimen of 300 repetitions could be tested in Phase IIb dose finding trial. Phase IIb trials test for a potential dose response relationship, or the optimal dose regimen (12). For example, a dose response trial would aim to evaluate the trend in performance of participants that receive four different doses (18), whereas a trial addressing the optimal dose would be powered to identify which dose is most effective (20).

### The Review and Design of the Tools to Examine Preclinical and Clinical Dose Articulation

To translate dose articulation findings between preclinical and clinical research we need to conduct systematic reviews that conceptualize the two collective bodies of research together. The starting point for the topic of dose articulation is a scoping review. To guide the reporting of a systematic scoping review, the Preferred Reporting Items for Systematic Reviews and Meta-Analysis extension for Scoping Reviews (PRISMA-ScR) is required (43). The PRISMA-ScR checklist states that included studies must be critically appraised and synthesized (43). To critique included studies across the discovery pipeline in this review, preclinical and clinical dose articulation research needs to be synthesized. We have not identified a precedent for a review that collectively appraises both preclinical experiments and clinical studies. We cannot rely on a single available tool to assess intervention description, risk of bias, and study quality across the discovery research pipeline. The tools used to address these concepts will be discussed below.

#### Intervention Description Tools

The extraction of key information on the reported dose constructs (time, scheduling, and intensity), and how they are articulated in the included studies is essential to address the review aims. Figure 1D outlines pre-existing intervention description tools that will be used to extract the required dose information. Data from the Animal Research: Reporting of *in vivo* Experiments (ARRIVE) (21) and Template for Intervention Description and Replication (TIDieR) Checklist (22, 44) will be synthesized to understand how dose is currently articulated, and whether the multidimensional constructs are appropriately reflected in stroke recovery research. The TIDieR tool is considered to be adequately suited to the task, however enhancement to the ARRIVE checklist to address the translation of findings from animals to humans is necessary.

Construct validity captures the degree to which preclinical findings can be generalized to the clinical population. The ARRIVE tool provides sufficient guidance to broadly address the translation, but there is no guidance on how it can be operationalized for stroke recovery research specifically. Five of the original ARRIVE questions were adapted to include specific prompts to extract information on the construct validity of included preclinical stroke experiments. For example, a prompt was added to the “*generalisability and translation*” question to extract information on whether the animal infarct size was proportional to what might be seen in a human stroke patient. Information collated from ARRIVE and TIDieR provides detailed insights into how dose is currently articulated and supports the discussion to build a discovery pipeline that prepares the dose to be translated from animal experiments to human studies.

#### Risk of Bias Tools

Type of bias varies across the discovery pipeline. The following tools were chosen as they examine the risk of bias specific to the field (preclinical and clinical) and design (non-randomized and randomized) of the included studies. For example, a Phase I trial should purposefully sample to ensure the dose ranging result reflects the performance of the target population (selection bias) but the completion of blinded outcome measures (detection bias) is less relevant as the goal of the design is not to determine causality. The pre-existing risk of bias tools are outlined in Figure 1E and include, Systematic Review Center for Laboratory Animal Experimentation's Risk of Bias Tool (SYRCLE-RoB) (23), Risk of Bias in Non-Randomized Studies—of Interventions (ROBINS-I) (25), and Cochrane Risk of Bias tool 2 (Cochrane RoB 2) (24). The SYRCLE-RoB tool was developed based on the Cochrane RoB tool and allows consistency of extracted information across the discovery pipeline.

#### The Development of a New Quality Tool

The development of a new quality tool capable of collectively appraising the dose articulation aspects of included preclinical and clinical studies is required. There is no quality assessment tool available on the EQUATOR Network (45) to address this requirement. A quality assessment checklist for Phase I cancer trials (26) was identified as the most relevant tool available to address the question of dose in pharmaceutical trials. This tool was used as a reference point to design a new tool called *Early Phase Research Quality Checklist (EPRQC)*, highlighted in Figure 1F. The final version of the tool can be found in Table 1. The checklist was adapted for application to (a) preclinical experiments and clinical early phase trials (Phase I to IIb) and (b) stroke recovery research rather than drug research. The initial aim for the checklist was to have consistent questions across all phases of the discovery pipeline. This could not be achieved due to the diversity of research designs that are required to specifically target each phase. For example, the application of dose limiting criteria to a Phase I dose ranging trial is critical to identify the maximum dose that is safe and tolerable. This design is not feasible in preclinical experiments as animals need to be motivated by food to participate in rehabilitation e.g., the concept of applying dose limiting criteria may reflect the point of satiation for the animals rather than the maximum dose.

**Table 1:** Early phase research quality checklist.

Early Phase Research Quality Checklist: EPRQC^*^						
**REFERENCE**						
**Phase**	**Preclinical**	**Clinical—Phase I**	**Clinical—Phase IIa**	**Clinical—Phase IIb**	**Clinical—Phase IIb**	**Reported on page no, comments**
	**Dose preparation**	**Dose ranging**	**Dose screening**	**Dose finding (response)**	**Dose finding (optimal)**	
Aim	To investigate the response to systematic variations of individual dose constructs.	To systematically escalate and de-escalate dose to identify minimum to maximum tolerated dose range.	To screen a dose regimen to determine if it is sufficiently promising to test in a phase IIb trial; considering feasibility, safety, and efficacy.	To investigate a potential dose response relationship of a dose regimen (includes single and/or multiple doses).	To identify the optimal dose regimen to test in a Phase III trial.	
**OBJECTIVE**						
1	Was one of the experiment objectives to investigate the response to individual dose construct?	Was one of the study objectives to find a dose range (minimum to maximum dose)?	Was one of the study objectives to screen a dose/s?	Was one of the study objectives to investigate the response to a dose regimen?	Was one of the study objectives to identify the optimal dose regimen?	
2a	N/A	Was there a prespecified list of “dose limiting criteria”?	N/A	N/A	N/A	
2b	N/A	Was there a limiting value assigned to the “dose limiting criteria”?	N/A	N/A	N/A	
3	N/A	Did the study differentiate between “dose limiting criteria” and events related to underlying disease, and/or unrelated adverse events?	Did the study differentiate between causality related adverse events and underlying disease progression, and/or unrelated adverse events?	Did the study differentiate between causality related adverse events and underlying disease progression, and/or unrelated adverse events?	Did the study differentiate between causality related adverse events and underlying disease progression, and/or unrelated adverse events?	
4	Was the chosen measure/s appropriate to test the targeted outcome and was it translatable to the clinical population?	Was a justification for the “dose limiting criteria” provided, and were the measures valid and reliable?	Was a justification for the chosen measure/s provided, and were the measures valid and reliable?	Was a justification for the chosen measure/s provided, and were the measures valid and reliable?	Was a justification for the chosen measure/s provided, and were the measures valid and reliable?	
**DESIGN: DOSE SPECIFICATION**						
5a	N/A	Was the starting dose specified?	Was the dose to be screened specified?	Was the lowest dose within the dose regimen specified?	Was the lowest dose within the dose regimen specified?	
5b	N/A	Was the starting dose justified?	Was dose to be screened justified?	Was the lowest dose within the dose regimen justified?	Was the lowest dose within the dose regimen justified?	
6a	Was the dose regimen clearly outlined?	Did the study state how the dose level will be determined for the second and subsequent cohorts?	N/A	Was the dose regimen clearly outlined?	Was the dose regimen clearly outlined?	
6b	Was the dose regimen justified?	Was a justification provided for how the dose levels were determined?	N/A	Was the dose regimen justified?	Was the dose regimen justified?	
7	Was the dose allocation method clearly described?	Was the dose allocation method clearly described?	Was the dose assignment method clearly described?	Was the dose allocation method clearly described?	Was the dose allocation method clearly described?	
**ANALYSIS**						
8	Was the definition of the data analysis set appropriate for the study design?	Was the definition of the data analysis set appropriate for the study design?	Was the definition of the data analysis set appropriate for the study design?	Was the definition of the data analysis set appropriate for the study design?	Was the definition of the data analysis set appropriate for the study design?	
9	Was the actual dose regimen and responses clearly reported?	Was the actual dose range (minimum to maximum dose) and responses clearly reported?	Was the actual dose/s and response/s clearly reported?	Was the actual dose regimen and responses clearly reported?	Was the actual dose regimen and responses clearly reported?	
10	Did the dose allocation match the described methods?	Did the dose allocation match the described methods?	Did the dose assignment match the described methods?	Did the dose allocation match the described methods?	Did the dose allocation match the described methods?	
11	Was a rationale for the statistical method chosen provided?	Was a rationale for the statistical method chosen provided?	Was a rationale for the statistical method chosen provided?	Was a rationale for the statistical method chosen provided?	Was a rationale for the statistical method chosen provided?	
12	Was the process of estimating the recommended dose regimen clearly explained?	Was the process of estimating the dose range (minimum to maximum dose) to be tested in phase IIa trials clearly explained?	Was the process of estimating the recommended dose to test in phase IIb trials explained?	Was the process of estimating the response to the dose regimen to be tested in later phase IIb clinical trials explained?	Was the process of estimating the optimal dose regimen to be tested in phase III clinical trials explained?	
	Score	Score	Score	Score	Score	

*SCORING: N/A or Not relevant = 0. Relevant and stated in the article = 1. Relevant but not stated in the article = −1*.

* *This table has been adapted from the Phase I quality assessment checklist (26). For full details of the adaptation process see Supplementary Material 1*.

The diversity of research designs meant that removal of non-relevant questions was required for some phases. To ensure non-relevant questions are accounted for in the scoring, the following system will be implemented: non-relevant questions scored zero (0), relevant questions not reported by the study scored minus one (−1), and relevant questions reported by the study scored one (1). The adaptation process was completed by two authors (ED and KH). Feedback was sought from a biostatistician and methodologist (LC) and the preclinical and clinical domains were reviewed by researchers with expertise in each area (DC/NL). Pilot testing was completed with four studies, one from each phase of the discovery pathway. Feedback was combined, and a final version of the tool was approved by all authors. Supplementary Material 1 online details the adaptation process.

### Summary

A barrier to progressing stroke recovery research is the failure to consider the multidimensional complexity of dose articulation, which informs how rehabilitation interventions are designed. A transparent and consistent approach to dose articulation across the discovery pipeline is required to strengthen stroke recovery research. To support the implementation of dose articulation across the discovery pipeline, we developed a new quality tool (EPQRC). The next section outlines our scoping review protocol, demonstrating the use of the EPRQC to address our specific research question.

## Methods and Analysis

To maintain the methodological rigor of the systematic scoping review, the PRISMA-ScR checklist (43) will be completed prior to publication. There is no Preferred Reporting Items for Systematic Reviews and Meta-Analysis Protocol (PRISMA-P) (46, 47) checklist available for a scoping review protocol. Compliance for this protocol, therefore, has been established through administration of PRISMA-P (developed for systematic reviews) with minor adaptations for relevance to the scoping review methodology. In this study we follow the scoping methodology described by Arksey and O'Malley (48) and include refinements which strengthen the rigor (49, 50). The process includes “*identifying the research question, identifying relevant studies, study selection, charting the data, and collating, summarizing and reporting the results*;” each of which is addressed below (48). The scoping review will be initiated on October 2019 and is expected to be completed by April 2020.

### Identifying the Research Question

Our main research question is *What is known about dose articulation in stroke recovery in both preclinical and clinical populations?*

Our specific aims are as follows:

1. Synthesize the literature on dose articulation in preclinical stroke recovery research;
2. Synthesize the literature on dose articulation in clinical stroke recovery research; and
3. Characterize the literature on research designs and statistical approaches used in dose articulation trials, and the associated advantages and disadvantages.

### Identifying Relevant Studies

Table 2 outlines the eligibility criteria. Consistent criteria across preclinical and clinical studies could not be achieved due to the different designs and language used. For example, in the clinical criteria we included the trial designs of dose ranging, screening, and finding for behavioral motor, stroke recovery interventions (upper limb, lower limb, exercise etc.). In the preclinical inclusion criteria, we had to target only upper limb rehabilitation (reaching or retrieval) post stroke due to the large volume (over 100,000) of returned studies in the pilot trial search strategy tested when we addressed stroke recovery broadly. Trial design was also removed from the preclinical criteria as they are not routinely reported in published experiments. To maximize the homogeneity of the included studies, preclinical experiments must test a dose regimen within their study (e.g., low training intensity vs. high training intensity or upper limb therapy).

**Table 2:** Eligibility criteria.

	**Aim 1: preclinical**	**Aim 2: clinical**
Inclusion	Study aims to articulate a dose regimen of an upper limb (reaching or retrieval), behavioral motor intervention	Study aims to articulate dose of a behavioral motor intervention through dose ranging, screening and finding designs
	Animals that have only had a focal ischemic or haemorrhagic stroke (100% of the sample has diagnosis of stroke within time frame)	Humans that have had only had an ischemic or haemorrhagic stroke (100% of the sample has diagnosis of stroke within time frame)
	Any stage of stroke recovery (a) Rodents; hyperacute (0–24 h), acute (1–5 days), early subacute (5 days−4 weeks), late subacute (30–60 days) or chronic (>60 days), (b) Non-human primate; hyperacute (0–24 h), acute (1–7 days), early subacute (7 days−6 weeks), late subacute (6 weeks−3 months), or chronic (>3 months) (5)	Any stage of stroke recovery; hyperacute (0–24 h), acute (1–7 days), early subacute (7 days−3 months), late subacute (3–6 months), or chronic (>6 months) (12)
		Adults >18 years old
Exclusion	Study aims to articulate dose of non-motor behavioral interventions (cognition, communication), drug or non-invasive brain stimulation (including those delivered in conjunction with motor behavioral interventions)	Study aims to articulate dose of non-motor behavioral interventions (cognition, communication), drug or non-invasive brain stimulation (including those delivered in conjunction with motor behavioral interventions)
	Study types: observational trials, scoping reviews, or systematic reviews	Study types: observational trials, qualitative trials, scoping review, or systematic review
Limits	English language Animals	English language Humans Adults (age ≥18)

Arksey and O'Malley's framework (48) supports the inclusion of studies with varying methodological quality. As such, observational studies will be excluded as they seek to understand the dose being provided under routine therapy conditions, rather than a systematic approach to articulating dose through dose ranging, screening, and finding designs. Both systematic and scoping reviews will be excluded as they are not empirical evidence. Hand searching of reference lists of relevant reviews and consultation with field experts will be completed to identify relevant literature.

### Study Selection

Systematic searches will be conducted in Medline and Embase. Two separate search strategies have been developed and use terms relevant to aim one (preclinical) and two (clinical). Each search strategy was designed using the Cochrane Database Search Strategies where applicable (e.g., stroke keywords) and further refined through consultation with a senior research librarian, preclinical (DC), and clinical (KH/NL) stroke recovery experts, and a biostatistician (LC). A draft of each search strategy can be found in online Supplementary Material 2.

Search results will be imported into Covidence (https://www.covidence.org/home) for transparent management (51). One author (ED) will screen titles and abstracts and remove duplicates as preliminary searches delivered a large volume of results. Two independent authors will review full text (ED/KH) and conflicts will be resolved by a third author who is an expert in either preclinical (DC) or clinical (NL) stroke recovery research, or design (LC). To maintain sound methodological processes, regular communication between authors throughout the selection process will occur (50). A PRISMA flow diagram will be used to track the screening process and any modifications (46).

### Data Charting

PRISMA-ScR recommends data charting as the most appropriate method of extracting data for scoping reviews (43). To adhere to the purpose of a scoping review, we developed two standardized electronic data forms to chart data from preclinical and clinical studies. Both forms have standardized extraction fields recommended for scoping reviews (49) and adhere to Cochrane's Checklist of items to consider in data collection and extraction where appropriate (52). Standard field categories include trial information, aim, population, methodology, intervention, and outcome. The intervention description tools discussed in the introduction, ARRIVE (21), and TIDieR (22, 44), will be included in the intervention category of the data extraction form. The risk of bias tools, SYRCLE-RoB (23), ROBINS-I (25), Cochrane RoB 2 (52), and the quality tool (EPRQC) will be listed in the outcome category. Figure 1 highlights how the tools will be implemented across preclinical and clinical studies, Figure 1D the intervention description tools, Figure 1E the risk of bias tools, and Figure 1F the quality tool. Online Supplementary Material 3 provides an example of the preclinical and clinical data extraction fields, focusing on understanding our research question of dose articulation.

The process of data extraction will be completed by one author (ED) with consistency verified by either a preclinical expert (DC) or clinical expert (KH/NL) by cross checking a random sample of 10% of included studies. The resulting findings extracted from the data will answer aim one and two of the scoping review.

### Collating, Summarizing, and Reporting of Results

The included trials will be collated in table form, summarizing the results of the above data charting process (aim one and two). A narrative synthesis will set the scene about current dose articulation literature, drawing out key conclusions. The findings from aim one and two will be characterized into key concepts of interest (aim three). A working conceptualization is research design, statistical approaches, and associated advantages and disadvantages of current dose articulation research. Understanding research design and statistical methods is the first step in undertaking systematic, high quality research. The purpose of this information is to help future researchers identify the most appropriate dose articulation design to answer their preclinical or clinical stroke recovery question.

## Discussion

The prospective publishing of this protocol is required to a priori state our intended aim and methods, as PROSPERO does not accept scoping reviews for registration. The unique features of this review include exploration of the multidimensional constructs of dose and the required implementation of a discovery pipeline in stroke recovery research as per SRRR consensus recommendations (9, 10). The focus of the proposed review is to establish a clear understanding of the articulation and development of dose clinical trials. This forms only one essential component of clinical trial development. It is important to note that within the complex system, which is a typical clinical trial, considerations of other components such as patient characteristics (e.g., body function) are also required (53).

The first step in supporting future research to adopt the discovery pipeline is the identification of current research gaps and breaking down the practice silos between the two fields. The development of the EPRQC addressed this gap and will be important to appraise the quality of dose articulation across preclinical and clinical literature. This tool will be useful for future research in this field and beyond. The identification of the “right dose” of rehabilitation through the systematic translation of findings across the pipeline will maximize the chance of future Phase III clinical trials showing clinically meaningful results. Although it is perhaps too early to fully appreciate the potential implications of the proposed research, we expect the findings from the proposed review will form part of the solution to understand what is the right dose of rehabilitation, at the right time, for the right person.

## Ethics Statement

Ethical approval is not required for this scoping review as we will use secondary de-identified data. The completed scoping review will form part of a Ph.D. thesis and be submitted for publication in a peer reviewed journal. Findings will be disseminated through presentations at appropriate conferences and other stroke recovery forums. The findings will be translated to support the completion of early phase, dose articulation trials in stroke recovery.

## Author Contributions

ED and KH conceived the idea for this review. ED, KH, and LC designed the Early Phase Research Quality Checklist (EPRQC). NL and DC provided critical review. ED led manuscript writing. All authors made significant intellectual contributions to the protocol and contributed to the drafting and editions, and approved the final manuscript.

### Conflict of Interest

The authors declare that the research was conducted in the absence of any commercial or financial relationships that could be construed as a potential conflict of interest.
