# Supplementary material for: Dose Articulation in Preclinical and Clinical Stroke Recovery: Refining a Discovery Research Pipeline and Presenting a Scoping Review Protocol
Source: Front Neurol. 2019 Nov 6;10:1148. doi: 10.3389/fneur.2019.01148 (PMC6851169; doi:10.3389/fneur.2019.01148)
Supplement: Supplementary file 1 [file Table_1.DOCX]

**Supplementary material 1: Early Phase Research Quality Checklist (EPRQC)– adaptation process**

| **Early Phase Research Quality Checklist (EPRQC): Adaptation process** | | | | | | | | |
| --- | --- | --- | --- | --- | --- | --- | --- | --- |
|  | **Domain** | **Zohar et al., 2008 (1)**  **Original question from Phase I quality checklist** | **Included**  **Y/N** | **Preclinical--**  **Dose Preparation** | **Clinical- Phase 1**  **Dose Ranging** | **Clinical- Phase IIa**  **Dose Screening** | **Clinical- Phase IIb**  **Dose Finding (response)** | **Clinical -Phase IIb**  **Dose Finding (optimal)** |
|  |  |  |  | To investigate the response to systematic variations of individual dose constructs | To systematically escalate and de-escalate dose to identify minimum to maximum tolerated dose range. | To screen a dose regimen to determine if it is sufficiently promising to test in a phase IIb trial; considering feasibility, safety and efficacy. | To investigate a potential dose response relationship of a dose regimen (includes single and/or multiple doses). | To identify the optimal dose regimen to test in a Phase III trial. |
| **1** | **Objective** | Was the objective to find a MTD or recommended dose for phase II trial? | Y | Was one of the experiment objectives to investigate the response to individual dose construct?  *E.g., Did the experiment compare the effective of no reaching, small dose of reaching or high dose of reaching in animals?* | Was one of the study objectives to find a dose range (minimum to maximum dose)?  *E.g., Did the study determine that 241 repetitions was the maximum dose of upper limb therapy achieved?* | Was one of the study objectives to explore a recommended dose/s?  *E.g., Was the feasibility of 400 repetitions of upper limb practice tested in post stroke patients?* | Was one of the study objectives to investigate the response to a dose regimen?  *E.g., Did the study investigated the response of participants who complete 200 reps vs 300 reps vs 400 reps of upper limb therapy?* | Was one of the study objectives to identify the optimal dose regimen?  *E.g., Did the study aim to identify that 500 repetitions was the most effective dose to give when compared to 400, 300 or 200 repetitions?* |
| **2a** | **Objective** | Was the MTD associated with a pre-specified toxicity rate? | Y | N/A | Was there a prespecified list of ‘dose limited criteria’?  *E.g., Did the study list the dose limiting criteria?* | N/A | N/A | N/A |
| **2b** |  | Additional question |  | N/A | Was there a limiting value assigned to the ‘dose limiting criteria’?  *E.g., Failure to complete more than 20% of prescribed weekly exercise dose due to pain.* | N/A | *N/A* | N/A |
| **3** | **Objective** | Was the toxicity related to the drug only used to define the MTD? | Y | N/A | Did the study differentiate between ‘dose limiting criteria’ and events related to underlying disease and/or unrelated adverse events?  *E.g., If the patient was unable to reach a dose level was it due to that dose or due to an event unrelated to the study such as a fall in the community?* | Did the study differentiate between causality related adverse events and underlying diseases and/or unrelated adverse events?  *E.g., If the patient only attended 50% of the sessions, did the authors provide a rationale for how they would determine if this was because of the dose intervention or because of the unrelated event?* | Did the study differentiate between causality related adverse events and underlying diseases and/or unrelated adverse events?  *E.g., If the patient scored a 9 on the pain visual analogue scale, did the authors provide a rationale for how they would determine if this was because of the dose regime or because of an unrelated event?* | Did the study differentiate between causality related adverse events and underlying diseases and/or unrelated adverse events?  *E.g., If the patient scored a 9 on the pain visual analogue scale, did the authors provide a rationale for how they would determine if this was because of the dose regime or because of an unrelated event?* |
| **4** | **Objective** | Was the measure of toxicity based on international grading system such as OMS or NCI scales? | Y | Was the chosen measure/s appropriate to test the targeted outcome and was it translatable to the clinical population?  *E.g., If forelimb recovery post stroke was the objective, did the experiment an appropriate outcome measure (staircase task) that allows for the translation to a human clinical trial* | Was a justification for the ‘dose limiting criteria’ provided, and were the measures valid and reliable?  *E.g., Was justification provided about how the dose limiting criteria was decided and then was it measured in a valid and reliable way?* | Was a justification for the chosen measure/s provided, and were the measures valid and reliable?  *E.g., Were the outcome measures justified from previous research and were they valid and reliable?* | Was a justification for the chosen measure/s provided, and were the measures valid and reliable?  *E.g., Were the outcome measures justified from previous research and were they valid and reliable?* | Was a justification for the chosen measure/s provided, and were the measures valid and reliable?  *E.g., Were the outcome measures justified from previous research and were they valid and reliable?* |
| **5** | **Objective** | Was the trial disease oriented? | N | Not relevant to stroke rehabilitation as targets the molecular elements specific to cancer medical research and therefore question will be dropped. | | | | |
| **6a** | **Design-**  **Dose Specification** | Was the starting dose specified? | Y | N/A | Was the starting dose specified?  *E.g., Statement that indicates starting dose = 15mins of task specific upper limb therapy.* | Was the dose to be screened specified?  *E.g., Statement that indicates the dose of 2 hours of task specific upper limb training will be screened.* | Was the lowest dose within the dose regimen specified?  *E.g., Statement that 1 hour of therapy will be the lowest dose in the regimen.* | Was the lowest dose within the dose regimen specified?  *E.g., Statement that 1 hour of therapy will be the lowest dose in the regimen.* |
| **6b** | **Design-**  **Dose Specification** | No question from Zohar et al., 2008 |  | N/A | Was the starting dose justified?  *E.g., Was the 15mins justified from other published research or pragmatic/clinical justification?* | Was dose to be screened justified?  *E.g., Was the 2 hours justified from other published research or pragmatic/clinical justification?* | Was the lowest dose within the dose regimen justified?  *E.g., Was the 1 hour justified from other published research or pragmatic/clinical justification?* | Was the lowest dose within the dose regimen justified?  *E.g., Was the 1 hour justified from other published research or pragmatic/clinical justification?* |
| **7a** | **Design-**  **Dose Specification** | Was the first dose level justified from preclinical or clinical data? | Y | Was the dose regimen clearly outlined?  *E.g., The low intensity group will complete 100 reaches, the high intensity group with complete 300 reaches and the control group will receive no therapy.* | Did the study state how the dose level will be determined for the second and subsequent cohorts?  *E.g., Was there a statement that the second and subsequent cohort will receive an additional 15mins of therapy until the dose limiting criteria is met?* | N/A | Was the dose regimen clearly outlined?  *E.g., This study will compare 100 reps to 200 reps to 300 reps of upper limb therapy to usual care.* | Was the dose regimen clearly outlined?  *E.g., This study will determine whether 600 reps per day is more effective than 300 reps her day of upper limb therapy.* |
| **8** | **Design-**  **Dose Specification** | Was the number of dose levels clearly mentioned in the study design? | Y | Was the dose regimen justified?  *E.g., Was an explanation provided for why this particular dose regimen is being tested?* | Was the justification provided for how the dose levels were determined?  *E.g., Was an explanation provided for why 15mins was chosen as the dose level?* | N/A | Was the dose regimen justified?  *E.g., Was an explanation provided for why this particular dose regimen is being tested?* | Was the dose regimen justified?  *E.g., Was an explanation provided for why this particular dose regimen is being tested?* |
| **9** | **Design-**  **Dose Specification** | Did the choice of the distinct levels explained? | N | Question dropped as the question was adapted and included in question 8 | | | | |
| **10** | **Design-**  **Dose Specification** | Was the dose allocation method clearly described? | Y | Was the dose allocation method clearly described?  *E.g., Were the animals randomised to particular groups or was there another method of allocation?* | Was the dose allocation method clearly described?  *E.g., Were the participants allocated to the next available cohort at time of consent?* | Was the dose assignment method clearly described?  *E.g., Were participants were allocated at time on consent?* | Was the dose allocation method clearly described?  *E.g., Were the participants randomised to particular groups or was there another method of allocation?* | Was the dose allocation method clearly described?  *E.g., Were the participants randomised to particular groups or was there another method of allocation?* |
| **11** | **Analysis** | Were all included participants analyzed? | Y | Was the definition of the data analysis set appropriate for the study design?  *E.g., Were all animals included in the analysis?* | Was the definition of the data analysis set appropriate for the study design?  *E.g., If appropriate, were all participants included in the analysis?* | Was the definition of the data analysis set appropriate for the study design?  *E.g., If appropriate, were all participants included in the analysis?* | Was the definition of the data analysis set appropriate for the study design?  *E.g., If appropriate, were all participants included in the analysis?* | Was the definition of the data analysis set appropriate for the study design?  *E.g., If appropriate, were all participants included in the analysis?* |
| **12** | **Analysis** | Were doses and responses clearly reported? | Y | Was the actual dose regimen and responses clearly reported?  *E.g., A table provided to outline the total number of reaches per animal and their response during the experiment.* | Was the actual dose range (minimum to maximum dose) and responses clearly reported?  *E.g., At table provided to outline the total time on task for each participant and their response during the experiment.* | Was the actual dose and response clearly reported?  *E.g., A table provided to outline the number of repetitions completed for each participant and their response during the experiment.* | Was the actual dose regimen and responses clearly reported?  *E.g., A table provided to outline the number of repetitions completed for each participant and their response during the experiment.* | Was the actual dose regimen and responses clearly reported?  *E.g., A table provided to outline the number of repetitions completed for each participant and their response during the experiment.* |
| **13** | **Analysis** | Did the dose allocation along the trial match the described methods? | Y | Did the dose allocation match the described methods?  *E.g., If the methods stated the animals would be randomised to dose groups, did this occur?* | Did the dose allocation match the described methods?  *E.g., If the methods stated that the participants would be allocated to the next available cohort at time of consent, did this occur?* | Did the dose assignment match the described methods?  *E.g., If the methods stated the participants would be randomised to dose groups, did this occur?* | Did the dose allocation match the described methods?  *E.g., If the methods stated the participants would be randomised to dose groups, did this occur?* | Did the dose allocation match the described methods?  *E.g., If the methods stated the participants would be randomised to dose groups, did this occur?* |
| **14** | **Analysis** | Was the estimated MTD or the recommended dose level associated with a measure of variability? | Y | Was a rationale for the statistical method chosen provided?  *E.g., Did the authors provide a rationale for why the statistical analysis plan was chosen?* | Was a rationale for the statistical method chosen provided?  *E.g., Did the authors provide a rationale for why the statistical analysis plan was chosen?* | Was a rationale for the statistical method chosen provided?  *E.g., Did the authors provide a rationale for why the statistical analysis plan was chosen?* | Was a rationale for the statistical method chosen provided?  *E.g., Did the authors provide a rationale for why the statistical analysis plan was chosen?* | Was a rationale for the statistical method chosen provided?  *E.g., Did the authors provide a rationale for why the statistical analysis plan was chosen?* |
| **15** | **Analysis** | Was the process of estimating the MTD or recommended the dose for future trials clearly explained? | Y | Was the process of estimating the recommended dose regimen clearly explained?  *E.g., 300 reps was more beneficial than 100 repetitions for stroke recovery in rats.* | Was the process of estimating the dose range (minimum to maximum dose) to be tested in phase IIa trials clearly explained?  *E.g., 90mins of time on task therapy was the maximum dose of upper limb therapy.* | Was the process of estimating the recommended dose to test in phase IIb trials explained?  *E.g., 600 reps per session for community stroke survivors was both feasible and demonstrated a positive trend to improving upper limb function.* | Was the process of estimating the response to the dose regimen to be tested in later phase IIb clinical trials explained?  *E.g., 2 hours of time on task upper limb therapy demonstrated a positive trend to recovery when compared to 1 hour or 30mins of therapy.* | Was the process of estimating the optimal dose regimen to be tested in phase III clinical trials explained?  *E.g., 2 hours of time on task upper limb therapy was more effective than 1 hour or 30mins of therapy* |

**References**

1. Zohar S, Lian Q, Levy V, Cheung K, Ivanova A, Chevret S. Quality assessment of phase I dose-finding cancer trials: proposal of a checklist. Clinical Trials. 2008;5(5):478-85.
