# Supplementary material for: Dose Articulation in Preclinical and Clinical Stroke Recovery: Refining a Discovery Research Pipeline and Presenting a Scoping Review Protocol
Source: Front Neurol. 2019 Nov 6;10:1148. doi: 10.3389/fneur.2019.01148 (PMC6851169; doi:10.3389/fneur.2019.01148)
Supplement: Supplementary file 2 [file Table_2.DOCX]

**Supplementary material 2: Example search strategy for scoping review**

| **Aim 1: Synthesis the literature on dose articulation in preclinical stroke recovery** | | | |
| --- | --- | --- | --- |
| **Stroke** | **Recovery** | **Intervention** | **Animals** |
| SH: Stroke  (stroke OR “cerebral infarct” OR “cerebrovascular accident” OR CVA OR “brain ischemia” OR “focal cortical ischemia” OR “cortical infarct” OR “cerebral ischemia” OR “MCA occlusion” OR “middle cerebral artery occlusion” OR “corticospinal tract”) | (recover* OR “recovery of function” OR “motor recover*” OR rehab* OR habilitat* OR “motor learn*” OR “motor control” OR “motor train*” OR train* OR intensity* OR “motor activit*” OR activit* OR behaviour OR behavior OR “treatment outcome”) | (reach* OR retrieval*)  (pellet adj3 (reach* OR retrieval*) | (animal OR mice OR rat OR “animal model” OR primate OR “non human primate”) |
| **Limits:** English | | | |

| **Aim 2: Synthesis the literature on dose articulation in clinical stroke recovery** | | |
| --- | --- | --- |
| **Stroke Recovery** | **Stroke** | **Trial Designs** |
| SH: Stroke rehabilitation  (recover* OR rehab OR rehabilitat* OR habilitat* OR “occupational therapy” OR physiotherap* OR “physical therapy modalit*” OR exercise* OR “exercise therap*” OR “physical therap*” OR “recovery of function” OR train* OR activit* OR behaviour* OR behavior* OR intensit* OR “task specific training*”) | SH: Stroke (cerebrovascular accident for Embase)  Stroke  (poststroke or “brain vasc*” OR “cerebral vasc*” OR apoplex* OR SAH)  ((brain* OR cerebr* OR cerebell* OR intracran* OR intracerebral OR vertebrovasilar) adj5 (isch?emi* OR infarct* OR thrombo* OR emboli* OR occlus*))  ((brain* OR cerebr* OR cerebell* OR intracerebral OR intracranial OR subarachnoid) adj5 (haemorrhage* OR haemorrhage* OR haematoma* OR hematoma* OR bleed*)) | ((dose OR dosage) adj3 (escalat* OR find OR finding OR respons* OR relationship*))  ((“early phase” OR “phase 0” OR “phase 1” OR “phase i” OR “phase 2” OR “phase ii” OR “phase 2a” OR “phase 2b” OR dose OR safety OR efficacy OR titrat* OR ramp* OR feasibility OR pilot) adj4 (trial OR trials OR study OR studies OR experiment OR experiments)) |
| **Limits** – Humans, English, Adult | | |
