# Supplementary material for: Dose Articulation in Preclinical and Clinical Stroke Recovery: Refining a Discovery Research Pipeline and Presenting a Scoping Review Protocol
Source: Front Neurol. 2019 Nov 6;10:1148. doi: 10.3389/fneur.2019.01148 (PMC6851169; doi:10.3389/fneur.2019.01148)
Supplement: Supplementary file 3 [file Table_3.DOCX]

**Supplementary material 3: Example data extraction forms (preclinical and clinical) for the scoping review.**

| PRECLINICAL DATA EXTRACTION FORM EXPLANATION | | |
| --- | --- | --- |
| TRIAL INFORMATION | | |
| Reference |  | |
| Scoping review criteria | Was our scoping review inclusion and exclusion criteria met?   1. Yes 2. No | |
| Geographic location | What country/ies was the study completed in? | |
| POPULATION | | |
| Age | Reported age (months, years) of subjects | |
| Age restriction | Was there an age restriction for the subjects?   1. Yes, and state age range restriction 2. No | |
| Animal type | Animal type and model | |
| Reason | Stated reason for chosen animal type?   1. Yes, and stated reason 2. Not stated | |
| Time post stroke | How long post stroke were the rodents?   1. Hyperacute – 0-24 hours 2. Acute – 1-5 days 3. Early subacute – 5 days – 4 weeks 4. Late subacute – 30 – 60 days 5. Chronic - > 60 days   OR  How long post stroke were the non-human primates?   1. Hyperacute – 0-24 hours 2. Acute – 1-7 days 3. Early subacute – 7 days – 6 weeks 4. Late subacute – 6 weeks -3 months 5. Chronic - > 3 months | |
| Stroke severity | Stroke severity of animals?   - Yes, and tool to demonstrate stroke severity - Not stated | |
| *NOTE: Gender is included in the ARRIVE Experimental Animals section. Housing conditions (number of animals and environment) is included in the ARRIVE Experimental Procedures section and the presents or absence of food restrictions is included in the ARRIVE Housing and Husbandry section. | | |
| METHODS |  |  |
| Pre-experiment training | Did the animals undergo training prior to stroke induction?   1. Yes, provide description 2. No 3. Not stated | |
| Pre-experiment training dose | Was the pre-experiment training dose stated?   1. Yes, provide description e.g., time, sessions, repetitions 2. No | |
| Methods of dose collection | What were the methods of dose collection?  E.g., observation of repetitions, electronic counter, vide reporting, researcher reports | |
| *NOTE: Description of post stroke training and procedure of inducing stroke in animals are included in the ARRIVE Experiment Procedures section. Research design category is included in the ARRIVE study design. Statistical approach is included in the ARRIVE Statistical Methods section. | | |
| INTERVENTION |  |  |
| Dose terms | What were the dose terms used in the study?   - E.g., dose-response relationship, dose-matching, dose-curve, dose intensity | |
| Dose definition/s | Did the authors state a dose definition?   1. Yes, and provide definition 2. Not stated | |
| Unidimensional or multidimensional | Was the dose unidimensional or multidimensional?   1. Unidimensional dose is considered as only one dose construct of a behavioural motor intervention 2. Multidimensional dose is considered as one or more dose constructs of a behavioural motor intervention | |
| Type of dose | What was the type of dose construct/s were used in the study?   1. Time in minutes or hours on task or in therapy 2. Repetitions of movement (reaches/retrievals) 3. Frequency in number of sessions per day and per week e.g., 2 sessions per day, 5 days per week) 4. Intensity 5. Duration length of program e.g., 10 week | |
| Animal Research: Reporting of In Vivo Experiments (ARRIVE) | Completion of ARRIVE checklist   - Report on individual ARRIVE items - Report number of “yes’ items for study | |
| *NOTE: Description of the experiments is included in the ARRIVE Experimental Procedures section. Starting dose and justification are included in EPRQC. | | |
| OUTCOME |  |  |
| Link dose to outcome | Dose the paper link dose with outcomes?   - E.g. dose-response used as variable in analysis | |
| Results of link between dose and outcome | If the paper dose link dose with outcome then provide the key findings?   - E.g., participants tolerated 200 reps of upper limb therapy | |
| Other results | Were there any other results of importance?   - E.g., | |
| Systematic Review Centre for Laboratory Animal Experimentation’s Risk of Bias Tool (SYRCLE) | Completion of SYRCLE RoB   - Report individual SYRCLE for all elements - Report overall “yes” items for the study | |
| Early Phase Research Quality Checklist (EPRQC) | Quality checklist for all study designs   - Report on individual EPRQC items - Report number of “yes” items for study | |
| *NOTE: The sample size of each experiment is included in the ARRIVE result section. | | |
| The above categories and questions will be added to an excel spreadsheet to allow for the extraction of data from included preclinical experiments. | | |

| CLINICAL DATA EXTRACTION FORM EXPLANATION | | |
| --- | --- | --- |
| TRIAL INFORMATION | | |
| Reference |  |  |
| Scoping review criteria | Was our scoping review inclusion and exclusion criteria met?   1. Yes 2. No | |
| Geographic location | What country/ies was the study completed in? | |
| POPULATION | | |
| Age | Reported age (years) of participants | |
| Age restricted | Was there an age restriction for the participants?   1. Yes, and stated age range restriction 2. No | |
| Time post stroke | How long post stroke were the participations?   1. Hyperacute - 0-24 hours 2. Acute - 1-7 days 3. Early subacute - 7 days- 3 months 4. Late subacute - 3-6 months 5. Chronic - >6 months 6. Not stated | |
| Reason | Was there a reason for participates to be a particular “time post stroke”?   1. Yes, and reason 2. Not stated | |
| Stroke severity | Stroke severity of participants?   1. Yes, and tool to demonstrate stroke severity e.g., National Institute Health Stroke Scale (NIHSS) 2. Not stated | |
| First stroke | Was it the participants first stroke?   1. Yes 2. No 3. Not stated | |
| Upper limb impairment | Did the participants have to meet a certain level of UL movement?   1. Yes - e.g., participants must be able to open/close hand 2. No 3. Not stated | |
| Lower limb impairment | Did the participant have to meet a certain level of LL movement?   1. Yes - e.g., participants must be able to move ankle 2. No 3. Not stated | |
| Cognition Impairment | Did the participant have to meet a certain level of cognition?   1. Yes - e.g., participants must have a MMSE of >22 2. No 3. Not stated | |
| Perception Impairment | Did the participant have to meet a certain level of perception?   1. Yes - e.g., participants must have a score of less than <1 on perceptual NIHSS score 2. No 3. Not stated | |
| Psychological Impairment | Did the participant have to meet a certain level of psychological state?   1. Yes - e.g., participants must not have a diagnosed psychological condition 2. No 3. Not stated | |
| Activity Impairment | Did the participant have to meet a certain level of function or activity?   1. Yes - e.g., participants must be able to walk 2. No 3. Not stated |  |
| Other | Did the participants have to meet a certain level on a criterion not defined above?   1. Yes 2. No 3. Not stated | |
| METHODS | | |
| Trial phase | Trial phase of study (definitions as per Australia Government Therapeutic Goods Administration).   1. Phase 0: “micro-dosing” which gathers preliminary data on whether a drug behaves as expected from preclinical trials 2. Phase I: “determines dosing” with an emphasis on “safety and tolerance” 3. Phase IIa: “clinical efficacy” through exploration of a therapeutic dose range 4. Phase IIb: “optimal therapeutic dose” with the aim to resolve design uncertainties for subsequent phase III trials | |
| Stated by authors | Was the trial phase stated in the methods?   1. Yes, and agrees with reviews allocation of trial phase 2. Yes, but is different to the reviewer’s allocation of trial phase 3. Not stated | |
| Methods of dose data collection | How was the dose information collected?   - E.g., observation by researches, self-report by participants, activity logs | |
| Primary outcome measure | Was there a primary outcome measure?   1. Yes - measure used e.g., ARAT 2. No – primary outcome was not stated | |
| Secondary outcome measure | What was the secondary outcome measure/s?   1. Yes - measure/s used e.g., FIM 2. Not stated | |
| Research design | Research design of the study?   - E.g., randomised control trial, feasibility study | |
| Statistical Approach | Statistical approach used on this study?   - E.g., 3+3 algorithm, ANOVA, t-tests | |
| INTERVENTION | | |
| Trial location | Where was the participant living location for the trial?   1. Home 2. Hospital 3. Both 4. Not stated | |
| Impairment targeted | Did the intervention tested target a specific impairment?   - E.g., wrist extension, grip strength | |
| Activity targeted | Did the intervention tested target a specific activity?   - E.g., Upper limb function or walking | |
| Dose terms | What were the dose terms used?   - E.g., dose-response relationship, dose-matching, dose-curve, dose finding | |
| Dose definition/s | Did the authors state a dose definition?   1. Yes, and provide definition 2. Not stated | |
| Dose articulation | How did the study articulate dose?   1. Dose Ranging 2. Dose Screening 3. Dose Finding (response) 4. Dose Finding (optimal) | |
| Unidimensional or multidimensional | Was the dose unidimensional or multidimensional?   1. Unidimensional dose is considered as only one dose construct of a behavioural motor intervention 2. Multidimensional dose is considered as one or more dose constructs of a behavioural motor intervention | |
| Type of dose | What was the type of dose construct/s were used in the study?   1. Time in minutes or hours on task or in therapy 2. Repetitions of movement e.g., wrist extension or function e.g., reaching to a cup 3. Frequency in number of sessions per day and per week e.g., 2 sessions per day, 5 days per week) 4. Intensity e.g., rating of perceived extension RPE 5. Duration length of program e.g., 10 week | |
| Template for Intervention Description and Replication Checklist (TIDieR) | Completion of TIDieR checklist   1. Report on individual TIDieR items 2. Report number of “yes’ items for study | |
| *NOTE: Location of therapy is included in the TIDieR (where). Starting dose, starting dose justification and dose incremental steps are included in the EPRQC. | | |
| OUTCOME | | |
| Sample size | Final sample size | |
| Link dose to outcome | Dose the paper link dose with outcomes?  E.g. dose-response used as variable in analysis | |
| Results of link between dose and outcome | If the paper links dose with outcome then provide the key findings?  E.g., participants tolerated 200 reps of upper limb therapy | |
| Other results | Were there any other results of importance?  E.g., participants tolerated 200 reps but no functional improvements noted | |
| Cochrane Risk of Bias tool (Cochrane RoB) | Risk of bias tool for randomised control trials   1. Report individual bias for each Cochrane RoB item 2. Report overall Cochrane RoB high, low or unclear | |
| Risk of Bias in Non-Randomised Studies – of Interventions (ROBINS-I) | Non-Randomised Studies – of Interventions (ROBINS-I) of non randomised trial designs.   1. Report individual biases for each ROBINS-I item 2. Report overall ROBINS-I low, moderate, serious, critical, no information | |
| Early Phase Research Quality Checklist (EPRQC) | Quality checklist for all study designs   1. Report on individual EPRQC items 2. Report number of “yes” items for study | |
| The above categories and questions will be added to an excel spreadsheet to allow for the extraction of data from included clinical trials. | | |
